# ARC^AgRP/NPY^ Neuron Activity Is Required for Acute Exercise-Induced Food Intake in Un-Trained Mice

**DOI:** 10.3389/fphys.2020.00411

**Published:** 2020-05-06

**Authors:** Wyatt Bunner, Taylor Landry, Brenton Thomas Laing, Peixin Li, Zhijian Rao, Yuan Yuan, Hu Huang

**Affiliations:** ^1^East Carolina Diabetes and Obesity Institute, East Carolina University, Greenville, NC, United States; ^2^Human Performance Laboratory, College of Health and Human Performance, East Carolina University, Greenville, NC, United States; ^3^Department of Kinesiology, East Carolina University, Greenville, NC, United States; ^4^Department of Physiology, East Carolina University, Greenville, NC, United States

**Keywords:** acute exercise, AgRP/NPY neuron, food intake, energy expenditure, metabolism

## Abstract

While much is known about the role of agouti-regulated peptide/neuropeptide Y (AgRP/NPY) and pro-opiomelanocortin (POMC) neurons to regulate energy homeostasis, little is known about how forced energy expenditure, such as exercise, modulates these neurons and if these neurons are involved in post-exercise feeding behaviors. We utilized multiple mouse models to investigate the effects of acute, moderate-intensity exercise on food intake and neuronal activity in the arcuate nucleus (ARC) of the hypothalamus. NPY-GFP reporter mice were utilized for immunohistochemistry and patch-clamp electrophysiology experiments investigating neuronal activation immediately after acute treadmill exercise. Additionally, ARC^AgRP/NPY^ neuron inhibition was performed using the Designer Receptors Exclusively Activated by Designer Drugs (DREADD) system in AgRP-Cre transgenic mice to investigate the importance of AgRP/NPY neurons in post-exercise feeding behaviors. Our experiments revealed that acute moderate-intensity exercise significantly increased food intake, ARC^AgRP/NPY^ neuron activation, and PVN^Sim1^ neuron activation, while having no effect on ARC^POMC^ neurons. Strikingly, this exercise-induced refeeding was completely abolished when ARC^AgRP/NPY^ neuron activity was inhibited. While acute exercise also increased PVN^Sim1^ neuron activity, inhibition of ARC^AgRP/NPY^ neurons had no effect on PVN^Sim1^ neuronal activation. Overall, our results reveal that ARC^AgRP/NPY^ activation is required for acute exercise induced food intake in mice, thus providing insight into the critical role of ARC^AgRP/NPY^ neurons in maintaining energy homeostasis in cases of exercise-mediated energy deficit.

## Introduction

The hypothalamus in the CNS is critical to the control of energy homeostasis, with the arcuate nucleus (ARC) and the sub-populations of neurons contained within it being especially important in fulfilling this role (Knner et al., 2009). The ARC contains two neuronal populations, which when activated, have opposite effects on feeding behavior: the anorexigenic pro-opiomelanocortin-expressing (POMC) neurons and the orexigenic agouti-related peptide/neuropeptide Y-expressing (AgRP/NPY) neurons. Activation of POMC neurons suppresses food intake (Santoso et al., 2017) by releasing α-melanocyte stimulating hormone (α-MSH), which binds to and activates melanocortin-4 receptors (MC4Rs) located at the paraventricular nucleus (PVN) (Balthasar et al., 2005). Furthermore, POMC knockout mice are over twice the weight of littermate controls at 3-months-old (Yaswen et al., 1999). Conversely, activation of AgRP/NPY neurons in the ARC by either chemical or optogenetic stimulation results in an immediate and robust increase in food intake (Aponte et al., 2011; Krashes et al., 2011), while ablation of AgRP/NPY neurons results in decreased food intake, which, if not reversed, can lead to starvation in adult mice (Gropp et al., 2005; Wu et al., 2009). These evidences highlight the novel but distinct roles of these two ARC neuron subpopulations in regulating food intake.

ARC^AgRP/NPY^ and ARC^POMC^ neurons can be regulated by changes in circulating factors and synaptic inputs as energy status fluctuates (Baskin et al., 1999). For example, during energy deficit, the stomach derived hormone, ghrelin, stimulates ARC^AgRP/NPY^ to promote food intake. In contrast, in the sated state leptin is released from adipose tissue to activate ARC^POMC^ and suppress food intake. Regarding neurocircuitry, the PVN is an important downstream site for the mediation of the ARC^POMC^ and ARC^AgRP/NPY^ regulation of energy homeostasis, with both subpopulations sending dense projections to this area (Wang et al., 2015). Lesions to the PVN and haploinsufficiency of Single-minded homolog 1 SIM1 (Leibowitz et al., 1981), which is expressed in the majority of PVN neurons, causes obesity, thus suggesting the ARC^AgRP/NPY^ → PVN^SIM1^ neurocircuitry is essential to the homeostatic regulation of food intake. However, the role of the ARC^AgRP/NPY^ → PVN^SIM1^ neurocircuitry in post-exercise feeding behaviors remains unclear.

Exercise significantly alters energy homeostasis by generating a temporary energy deficit. Surprisingly, studies investigating exercise as a weight-loss intervention elicit mixed success rates (Group, 2002; Stiegler and Cunliffe, 2006; King et al., 2008). Interestingly, documented compensatory eating post-exercise varies greatly across types, intensities, durations, and modes of exercise (Mayer et al., 1954; Kissileff et al., 1990; King et al., 1994; Verger et al., 1994; Stiegler and Cunliffe, 2006), possibly explaining discrepancies in studies using exercise as a weight-loss intervention. Further complicating our understanding of post-exercise feeding behavior, many commonly used animal models of exercise, including involuntary swimming and those using electricity as a motivator, are not physiologically relevant and induce additional stress confounding data. As a result, the purpose of this study was to investigate the effects of an acute, physiologically relevant, moderate-intensity, treadmill exercise protocol (≈ 75% VO2_Max_) on feeding behavior and ARC neuron activity to determine the physiological mechanisms involved. Furthermore, we determined the direct involvement of ARC neuron populations in post-exercise feeding behaviors using the Designer Receptors Exclusively Activated by Designer Drugs system (DREADD).

## Materials and Methods

### Experimental Animals

All animal procedures were approved by the Institutional Animal Care and Use Committee for the East Carolina University, Greenville, NC, United States. Two transgenic mouse lines were utilized; B6. Tg (NPY-hrGFP)1Lowl/J (NPY-GFP reporter) mice and the AgRP-Cre mouse model in which Cre recombinase expression is induced selectively in AgRP-expressing neurons. Mice were housed in a temperature-controlled environment (22–24°C) with a 12 h light (07:30)/dark (07:30) cycle with standard mouse chow and water provided *ad libitum*.

### Acute Treadmill Exercise Protocol

Mice were randomly assigned to an acute exercise or sedentary control group. On the day before experiments, all mice were familiarized by resting for 10 min on the treadmill followed by exercise for 5 min at 5 m/min and 5 min at 10 m/min. On the day of the experiment, exercise mice underwent a 10-minute stationary acclimation period on the treadmill, followed by 5 m/min for 2 min and then 13 m/min for an hour, which is estimated to be around 75% of VO2_max_ in adult mice (Schefer and Talan, 1996). At the same time, the mice in the sedentary group remained stationary on top of the treadmill apparatus for the same duration. For post-exercise food intake experiments, half the mice were assigned to an acute exercise group and half to the sedentary group, and a randomized crossover design was used with a week in between experiments. All conditions were maintained in the ARC^AgRP/NPY^ neuron inhibition experiments with the addition of either clozapine N-oxide (CNO) or saline injection 30 min prior to beginning the exercise protocol.

### Food Intake

Food intake was measured 0.5, 1, 2, 4, and 8 h immediately post-exercise. All mice were individually housed at least 1 week prior to exercise. Mice were placed in cages with alpha dry bedding 48 h prior to food intake measurements. Any residual food in the bedding was included in measurements. Cumulative food intake data was obtained by adding all intake measurements during the study.

### Glucose Measurements and Collection of Blood, Cerebrospinal Fluid (Csf), and Coronal Brain Sections

Immediately after the acute bout of exercise, mice were anesthetized with 99.9% isoflurane. In one subset of mice, blood samples were immediately collected via tail incision and blood glucose levels were measured (Relion Prime Blood Glucose Monitoring System, ARKRAY Inc., Kyoto, Japan). After blood glucose measurements, an incision was made in the neck of the mouse to expose the cisterna magna, a capillary tube was inserted through the dura mater to collect the CSF, and CSF glucose concentrations were measured. In another subset designated for immunohistochemical experiments, mice were intracardially perfused with phosphate-buffered saline (PBS) followed by 10% neutral buffered formalin. Brains were removed, stored in the same fixative for 24 h, and transferred into 30% sucrose at 4°C for at least 24 h. 20 μm coronal sections were generated and divided into five equal series using a freezing microtome (Leica VT1000 S) as previously described (Huang et al., 2012, 2013). Anatomical landmarks such as the median eminence and third ventricle were used to identify PVN and ARC sections, as well as the mouse brain atlas (Paxinos and Franklin’s the Mouse Brain in Stereotaxic Coordinates 4th Edition) and DAPI staining (H-1200, Vector Lab, Burlington, CA, United States).

### Immunohistochemistry

Brain sections were washed in PBS and blocked in 3% normal donkey serum in PBS+0.03% Triton (PBST) for 1 h at room temperature. Brain sections were then incubated overnight at room temperature in blocking solution containing primary antiserum (rabbit anti-POMC precursor, Phoenix Pharmaceuticals H-029-30, 1:3000; goat anti-Fos, Santa Cruz Biotechnology, sc-52-G, 1:500; rabbit anti dsRed for mCherry, Clontech, 1:1000; rabbit anti-SIM1, Millipore, 1:500). The next morning sections were extensively washed in PBS and then incubated in Alexa-fluorophore secondary antibody (A-21209, A-11039, both 1:500) for 1 h at room temperature. After several washes in PBS, sections were mounted on glass slides. For DAB (3,3′-Diaminobenzidine) staining, brain sections were washed and blocked in 3% normal donkey serum in PBST for 1 h at room temperature. Slices were then incubated in cfos goat primary antiserum (goat anti-FOS, Santa Cruz Biotechnology, sc-52-G, 1:1000) overnight followed by biotinylated donkey anti-goat IgG (Vector; 1:1000) for 2 h. Sections were then incubated in the avidin–biotin complex (ABC; Vector Elite Kit; 1:500) and incubated in 0.04% DAB and 0.02% cobalt chloride (Fisher Scientific), and 0.01% hydrogen peroxide. AgRP/NPY neurons were identified in sections by native fluorescence (green) of the green fluorescent protein (GFP) transgene from NPY-GFP Reporter mice. The sections were photographed digitally using an upright optical microscope (Leica DM6000, Wetzlar, Germany). 20x objectives were used to image either the left or the right hemisphere in the ARC of the hypothalamus. POMC, cfos, SIM1, DAPI, and NPY-positive neurons throughout the image were counted using ImageJ Cell Counter plug-in function for marking and numbering of positive cells. Once positive cells were marked, ImageJ software was used to overlay images to quantify colocalization. Three serial sections were double blind analyzed in each mouse (*n* = 3–5 mice per group).

### Stereotaxic AAV-HM4Di-mCherry Injections and the DREADD System

The DREADD system was used to insert a physiologically inert inhibitory G-coupled protein receptor specifically onto AgRP/NPY neurons. Briefly, using a stereotaxic device, 200 nL cre-dependent adeno-associated virus (AAV8.hSynp.hM4Di–mCherry, 8.3 × 10^12^ genomic copies per milliliter, Addgene, Watertown MA, United States) was injected bilaterally into the ARC of 5–6-week-old AgRP-Cre male mice (coordinates from bregma: anterior-posterior, −1.50 mm; dorsal-ventral −5.95 mm and −5.80 mm; lateral, ±0.20 mm) with a glass micropipette and air pressure injector system (Grass S48 Stimulator). After surgery, mice were individually housed and allowed 2 weeks to recover before the start of studies. Activation of these receptors and subsequent AgRP/NPY neuron inhibition was induced by intraperitoneal injection of CNO (0.3 mg/kg of body weight) (Krashes et al., 2011) or saline 30 min prior to the exercise or sedentary conditions. Validation of AAV expression and localization was performed by fluorescent microscopy visualization of mCherry.

### Electrophysiological Recordings

Immediately after the acute exercise, animals were deeply anesthetized, and intracardially perfused with an ice-cold N-methyl-D-glucamine (NMDG) solution consisting of (92 mM NMDG, 20 mM HEPES, 25 mM Glucose, 30 mM NaHCO3, 1.2 mM NaH2PO4, 2.5 mM KCl, 10 mM MgSO4, 0.5 mM CaCl, 5 mM sodium ascorbate, 3 mM sodium pyruvate, 2 mM Thiourea) measured osmolarity 310–320 mOsm/l, and decapitated. Brains were quickly removed into ice-cold NDMD solution, oxygenated with 95% O2/5% CO2, 300-μm-thick coronal sections were cut with a VF200 Compresstome (Precision Instruments, Greenville NC, United States) and incubated in oxygenated chilled for 10 min. Slices were transferred to oxygenated aCSF holding solution (92 mM NaCl, 20 mM HEPES, 25 mM Glucose, 30 mM NaHCO3, 1.2 mM NaH2PO4, 2.5 mM KCl, 10 mM MgSO4, 0.5 mM CaCl, 5 mM sodium ascorbate, 3 mM sodium pyruvate, and 2 mM Thiourea) and stored in the same solution at room temperature in a BSK 6 (Automate Scientific, Berkley CA, United States) (20–24°C) for at least 60 min before recording. A single slice was placed in the recording chamber where it was continuously perfused at a rate of 1–2 ml per min with oxygenated recording aCSF solution (125 mM NaCl, 11 mM Glucose, 26 mM NaHCO3, 1.25 mM NaH2PO4, 2.5 mM KCl, 10 mM MgSO4, 2.4 mM CaCl, and 1 mM MgCl). Neurons were visualized with an upright Leica DM6000F equipped with infrared differential interference contrast and fluorescence optics. Borosilicate glass microelectrodes (4–6 MΩ) were filled with internal solution. To assess the effect of exercise on ARC^AgRP/NPY^ neurons, a loose cell-attached recording (seal resistance >20 mΩ) were made in voltage clamp mode with potassium gluconate as internal solution and holding current maintained at Vh = −60 mV in NPY-GFP Reporter mice.

### Calculating Estimated Energy Expenditure and Excess Energy Consumption

To calculate the estimated energy expenditure of the mice undergoing the exercise treatment we used a previously validated equation for adult mice to predict their relative VO_2_ (ml/kg/hr) [VO_2_ = 5444 + (223 × Treadmill Velocity m/min.)] and then we performed indirect calorimetric calculations (Schefer and Talan, 1996).

To calculate the excess energy consumed in the exercise group compared to the sedentary group over the total time measured post-exercise (8 h), we subtracted the total weight in grams of the food consumed from each mouse during the exercise condition and subtracted this from the total food consumed during the sedentary condition. This difference was then multiplied by the metabolizable energy contained in their food source (3.20 Kcal/gram; Prolab Isopro RMH 3000).

### Statistical Analysis

Results are reported as the mean ± SEM. Statistical analyses were performed using Prism 6.0 (GraphPad) software. Food intake was analyzed by two-way repeated measures ANOVA with Sidak correction for multiple comparisons and *t*-test unpaired set to ^∗^*p* < 0.05 for significance. Static data such as total neurons, neurons co-localized with cfos, and firing rate was averaged and measured utilizing an unpaired *t*-test, and a one-way ANOVA with a Sidak correction when appropriate, with an alpha value set at 0.05 for significance. All patch clamp recordings were reported offline using Clampfit 10.6 to measure firing rate.

## Results

### An Acute Bout of Exercise Increases ARC^AgRP/NPY^ Neuron Activation While ARC^POMC^ Neuron Activation Remains Unchanged

**FIGURE 1:**
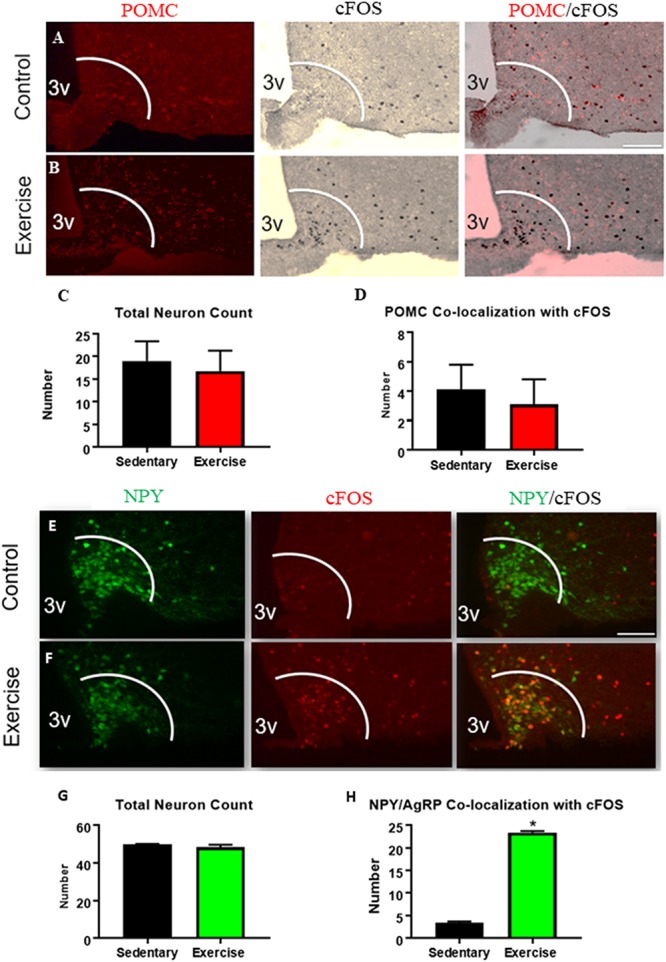
An acute bout of exercise increases ARC^AgRP/NPY^ neuron activation while ARC^POMC^ neuron activation remains unchanged. Expression of cfos in POMC-expressing neurons in ARC sections from **(A)** sedentary control and **(B)** exercise groups immediately after an acute bout of exercise. **(C)** Average POMC neuron count per slice. **(D)** Average number of neurons that are co-localized with cfos. Expression of cfos in ARC^AgRP/NPY^ neurons in **(E)** sedentary control and **(F)** exercise groups immediately after an acute bout of exercise. **(G)** Average ARC^AgRP/NPY^ neuron count per slice **(H)** ARC^AgRP/NPY^ neuron’s that are co-localized with cfos. 3V: third ventricle; scale bars represent 50 μm. Bar graphs show Mean + SEM. (*N* = 6 male mice per group), * indicates *p* < 0.05 vs. sedentary group.

There were no differences in the number or activation (cfos) of ARC^POMC^ expressing neurons in the exercise group compared to the sedentary group (Figures 1A–D). The number of ARC^AgRP/NPY^ neurons that were colocalized with cfos was significantly increased in the exercise group (23.3 ± 0.3 active ARC^AgRP/NPY^ neurons per slice) compared with the sedentary group (3.3 ± 0.3 active ARC^AgRP/NPY^ neurons per slice) (Figures 1E–H), indicating increased activation of ARC^AgRP/NPY^ neurons after an acute bout of exercise.

### ARC^AgRP/NPY^ Neuron Firing Rate *ex vivo* Is Increased After an Acute Bout of Exercise

**FIGURE 2:**
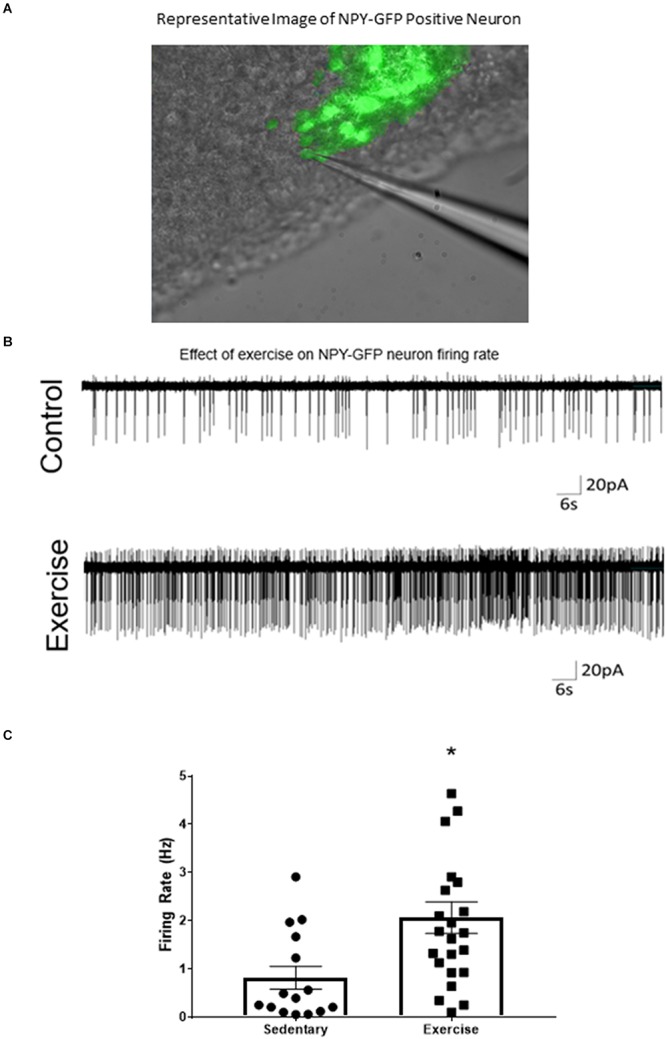
ARC^AgRP/NPY^ neuron firing rate *ex vivo* is increased after an acute bout of exercise. **(A)** Representative image of patch-clamp pipette sealed to NPY-GFP neuron in the ARC. **(B)** Representative cell attached trace of ARC^AgRP/NPY^ neuron firing rate in voltage clamp (–50 mV) after sedentary and exercise conditions. **(C)** Calculated firing rate of ARC^AgRP/NPY^ neurons in mice. Data are expressed as mean ± SEM. (*N* = 15–22 neurons from 8 mice (4 male and 4 female) per group) * indicates *p* < 0.05 vs. sedentary group.

A loose cell-attached voltage-clamp electrophysiology was used to measure the effects of acute exercise on ARC^AgRP/NPY^ neuron firing rate. There was a significant increase in firing rate of ARC^AgRP/NPY^ neurons immediately after exercise (2.07 ± 0.33 Hz) compared to the sedentary group (0.82 ± 0.24 Hz) (Figure 2).

### Food Intake Is Increased Immediately Post-exercise

**FIGURE 3:**
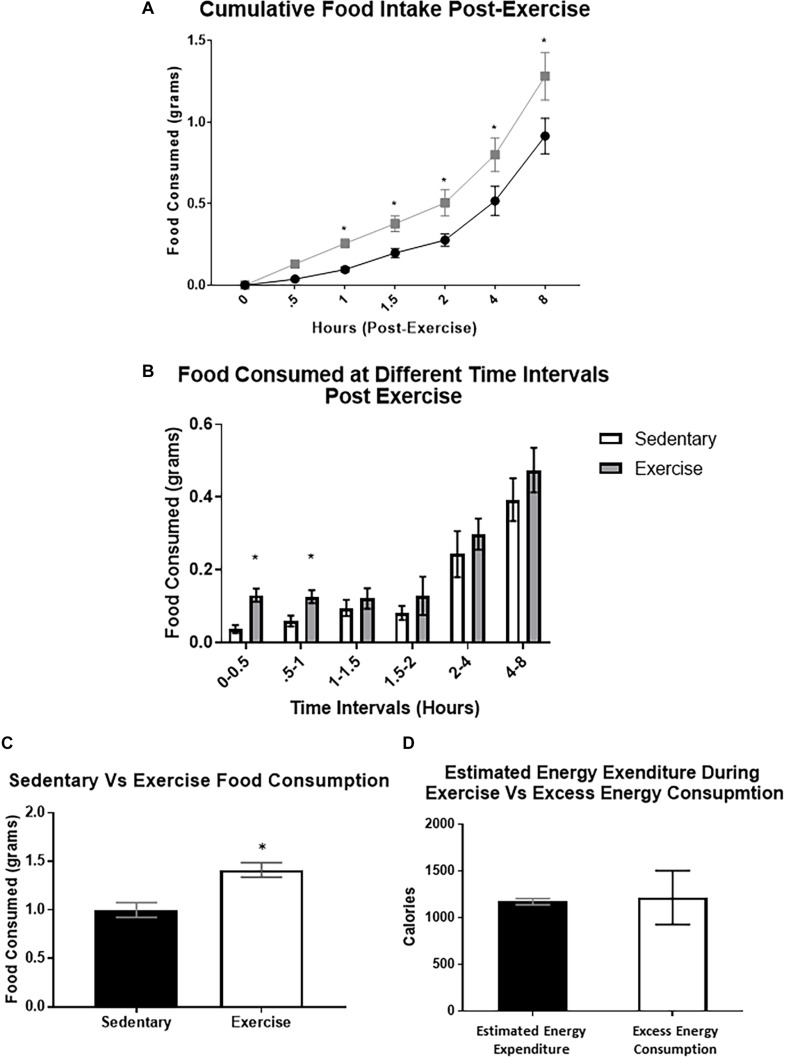
Food intake is increased immediately after a bout of moderate-intensity exercise. **(A)** Average cumulative food intake in mice over 8 h immediately after the sedentary or exercise conditions. **(B)** Average food consumed during specific time periods post-exercise **(C)** Total food consumed after 8-hour time period. **(D)** Estimated energy expenditure during the acute exercise bout compared to the excess caloric consumption (excess caloric consumption defined as calories consumed by the exercise group minus calories consumed by the sedentary group). Bar graphs show Mean + SEM. (*N* = 10 male mice per group), * indicates *p* < 0.05 vs. sedentary group.

After exercise, cumulative food intake was significantly increased compared to the sedentary group at the 1, 2, 4, and 8 h time points (cumulative 8-hour food intake was (1.41 ± 0.30 g) compared to 0.92 ± 0.11 g) (Figures 3A,C). When analyzing food intake during specific time intervals, food consumed was significantly elevated between 0–30 min and 30–60 min (Figure 3B). We also used a previously validated equation (Schefer and Talan, 1996) to compare estimated energy expenditure during the acute exercise bout with the excess caloric consumption (excess caloric consumption was defined as calories consumed by the exercise group minus calories consumed by the sedentary group). There was no significant difference between the two measures (Excess energy consumed: 1215 ± 288.8 Cal vs. estimated energy expenditure: 1172 ± 34.7 Cal), indicating that the excess energy intake may be compensating for the energy deficit caused by exercise (Figure 3D).

### Blood and CSF Glucose Levels Were Elevated Immediately Post-exercise

**FIGURE 4:**
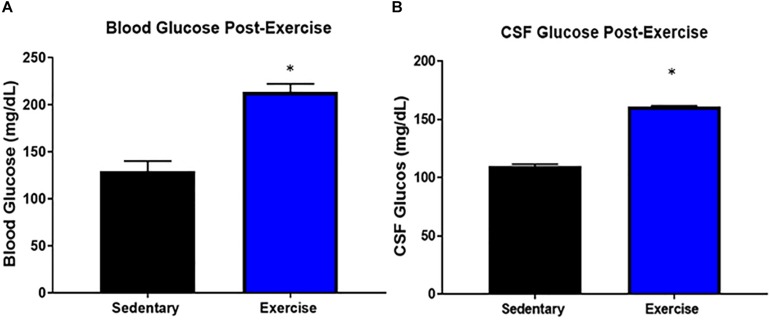
Blood and CSF glucose levels were elevated immediately post-exercise. Average circulating glucose levels in **(A)** blood and **(B)** CSF of mice immediately after a bout of acute moderate-intensity exercise. Bar graphs show Mean + SEM. (*N* = 3 male mice per group), * indicates *p* < 0.05 vs. sedentary group.

Both CSF (161.2 ± 0.7 mg/dL) and blood glucose levels (213.5 ± 8.5 mg/dL) were significantly increased in the exercise group compared to the control group (CSF glucose: 110 ± 1.4, blood glucose: 129.2 ± 10.8) immediately post-exercise (Figure 4).

### AgRP Neuron Inhibition Abolishes Acute Exercise Induced Food Intake

**FIGURE 5:**
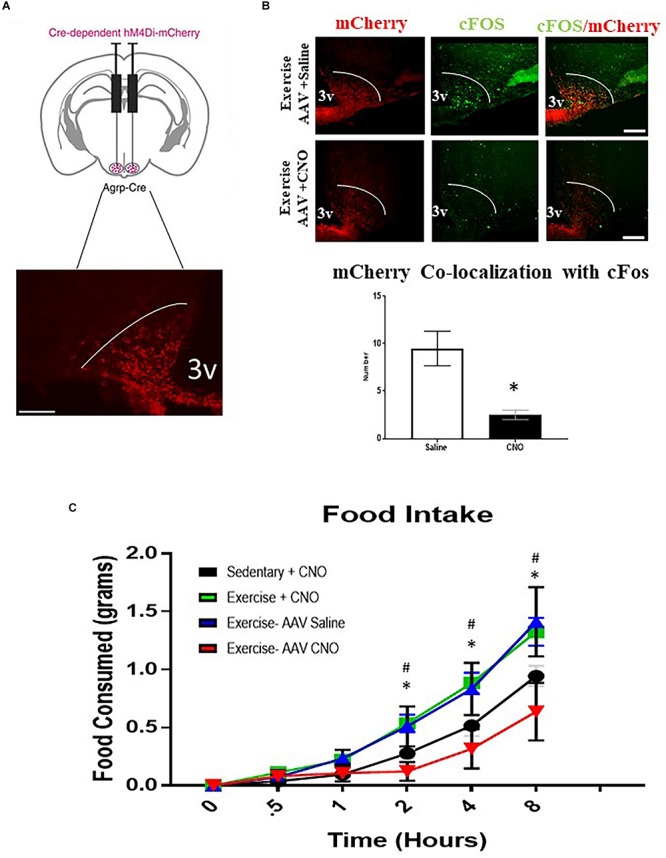
AgRP neuron inhibition abolishes acute exercise induced food intake. **(A)** Top, targeting scheme for hM4Di-mCherry. Bottom, localization of hM4Di-mCherry with the anatomical location of NPY/AgRP neurons in the ARC. **(B)** Immunofluorescence and quantification of mCherry positive and cfos-expressing cells in the ARC from AAV CNO and AAV Saline groups immediately after a bout of acute exercise. **(C)** Cumulative food intake over an 8-hour period immediately after the exercise or sedentary conditions. ^#^ indicates significance between Exercise CNO and Sedentary CNO groups (*p* < 0.05); * indicates a significant difference between Exercise AAV Saline and Exercise AAV CNO groups (*p* < 0.05) (*N* = 6 male mice per group). (I.P. CNO and saline injections of 0.3 mg/kg of body weight were applied 30 min prior to exercise). Bar graphs show Mean + SEM. 3V: third ventricle, scale bars represent 50 μm.

To determine whether ARC^AgRP/NPY^ neuron activity is required for acute exercise-induced food intake, the DREADD system was utilized in conjunction with AgRP-Cre transgenic mice. I.P. injection with saline or CNO (0.3 mg/kg of body weight) alone to non-DREADD treated mice before exercise had no effects on post-exercise food intake. Interestingly, DREADD treated mice receiving CNO injections to inhibit ARC^AgRP/NPY^ neuronal activation experienced significantly decreased food intake (0.64 ± 0.25 g 8 h post-exercise) compared to the exercise groups treated with CNO (1.28 ± 0.15 g) or saline alone (1.41 ± 0.30 g) (Figure 5).

### Acute Exercise Induces PVN^SIM1^ Expressing Neuronal Activation Independent of ARC^AgRP/NPY^ Neurons

**FIGURE 6:**
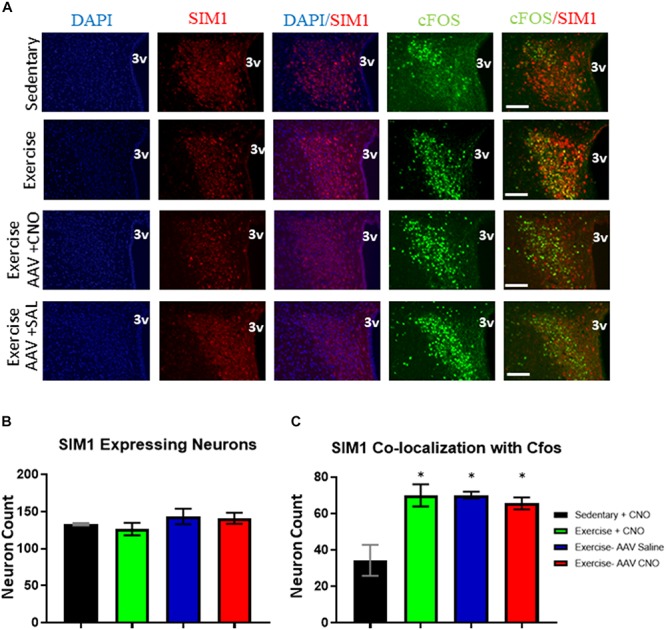
Acute exercise induces PVN^SIM1^ expressing neuronal activation independent of ARC^AgRP/NPY^ neurons. **(A)** Immunofluorescence of SIM1-positive and cfos-expressing cells in the PVN from the sedentary, exercise, AAV CNO, and AAV Saline groups immediately after a bout of acute exercise. 3V = third ventricle; scale bars represent 50 μM. **(B)** Quantification of SIM1-positive cells in the PVN among the four groups. **(C)** Quantification of cfos colocalization with SIM1-positive cells in the PVN from the sedentary, exercise, AAV CNO, and AAV Saline groups immediately after a bout of acute exercise. 3V: third ventricle, scale bars represent 50 μm. Bar graphs show Mean + SEM. (*N* = 6 from 3 male mice per group), * indicates *p* < 0.05 vs. sedentary group.

All exercise groups had increased cFos colocalization in PVN^SIM1^ expressing neurons, with the exercise, DREADD with CNO, and DREADD with saline groups averaging 70.0 ± 6.1, 70.2 ± 1.8, and 65.5 ± 5.6 active PVN^SIM1^ neuron colocalization with cFos per slice, respectively, compared to 34.3 ± 8.5 per slice colocalization in the sedentary group. There was no significant difference in the number of SIM1 expressing neurons between the sedentary, exercise, DREADD with saline, and DREADD with CNO groups (Figure 6). There were also no significant differences in cfos expression in PVN^SIM1^ neurons among the three exercise groups (Figure 6), suggesting ARC^AgRP/NPY^ activity is not involved in exercise-induced PVN^SIM1^ activity.

## Discussion

In this study we investigate the effect of an acute bout of exercise on the activity of ARC^AgRP/NPY^ and their adjacent ARC^POMC^-expressing neurons in the hypothalamus, as well as the role of ARC^AgRP/NPY^ neuron activation in the associated feeding response post-exercise. In NPY-GFP reporter mice, both immunostaining for cfos and electrophysiological recording revealed that acute moderate-intensity exercise increases ARC^AgRP/NPY^ neuron activity and has no effects on ARC^POMC^ neuron activation. Concurrently, food intake was significantly increased immediately after the acute bout of exercise compared to sedentary conditions. Furthermore, chemo-genetic inhibition of ARC^AgRP/NPY^ neurons completely abolished exercise-induced food intake, suggesting ARC^AgRP/NPY^ neurons are critically involved in refeeding after exercise.

ARC^AgRP/NPY^ neuron activation is normally induced by food restriction, but these neurons are also modulated by altered plasma hormone levels associated with stressful conditions, like exercise (Tong et al., 2008; Lin et al., 2010). Single bouts of exercise have been shown to alter circulating hormone levels such as ghrelin and insulin (Berger et al., 1975; Dey et al., 1992; Broom et al., 2009; Mani et al., 2018), and those hormones have been shown to modulate the activity of ARC^AgRP/NPY^ neurons (Björntorp, 1981; Xu et al., 2008; Mani et al., 2018). For example, acylated ghrelin is elevated during exercise, and deletion of ghrelin receptor (GHSR) markedly reduces post-exercise food intake (Mani et al., 2018). These data suggest the importance of exercise induced ghrelin signaling in the regulation of food intake (Mani et al., 2018), and future study is needed to investigate the possible direct role of ghrelin on post-exercise ARC^AgRP/NPY^ activity.

ARC^AgRP/NPY^ neurons also possess glucose-sensing properties, and, consistent with findings from other studies (Mani et al., 2018), both blood and CSF glucose levels were observed to be elevated immediately after the acute exercise bout. However, high glucose concentrations have been shown to inhibit ARC^AgRP/NPY^ neurons (Routh, 2010), indicating that glucose levels are unlikely the direct reason for increased ARC^AgRP/NPY^ activation post-exercise. Altered neurotransmitter release, such as glutamate (excitatory) and GABA (inhibitory), is also critical to the regulation of neuronal activity. It has been reported that acute exercise reduces GABA synaptic input onto neurons in the Nucleus Tractus Solitarii (NTS) to regulate blood pressure in rats (Chen et al., 2009). The NTS has well-documented synaptic connections with ARC neurons, thus, suggesting a pre-synaptic mechanism on which to focus future research into ARC^AgRP/NPY^ control of post-exercise refeeding (Andermann and Lowell, 2017).

ARC^AgRP/NPY^ neuron activity has been shown to decrease energy expenditure and promote food intake in response to states of energy deficit to maintain energy homeostasis (Aponte et al., 2011; Krashes et al., 2011). Thus, the increase in ARC^AgRP/NPY^ activation observed in this study, and subsequent increase in food intake, may suggest a potential compensatory mechanism to conserve energy during the energy deficit caused by exercise. Interestingly, when estimating the total energy expended by exercise using a previously validated equation (Schefer and Talan, 1996), we found that the energy expenditure during the acute bout of exercise is comparable to the additional caloric intake post-exercise. This may suggest a CNS-mediated compensatory mechanism in mice that promotes food intake after forced energy expenditure via moderate-intensity treadmill exercise to maintain energy homeostasis. Notably, this observed significant increase in food intake was predominantly evident in the first hour post-exercise, which is often the timeline observed for ARC^AgRP/NPY^ neuron activation to induce robust increases in food intake (Krashes et al., 2011). Furthermore, when ARC^AgRP/NPY^ neuron activation was inhibited through chemo-genetic inhibition, this acute exercise induced energy intake was completely abolished, indicating that ARC^AgRP/NPY^ neuronal activation is required for acute exercise induced food intake to maintain energy balance. Notably, a limitation of the DREADD system is off-target action due to possibility of Cre-Leakage, however, use of AAV and CNO controls in the current study suggest that this is unlikely.

The effect of exercise on food consumption has previously been studied in both humans and rodents. However, the observations have been controversial due to differences in the intensity, mode, duration, and volume of exercise performed (Mayer et al., 1954; Kissileff et al., 1990; King et al., 1994; Verger et al., 1994; Stiegler and Cunliffe, 2006). Contrary to the current study, in a recent publication using high intensity interval training in mice (>90% VO2_MAX_), it was reported that food intake was significantly decreased immediately after a single bout of acute exercise. Moreover, the resting membrane potentials of ARC^POMC^ neurons were increased, while an opposite effect was observed in ARC^AgRP/NPY^ neurons (He et al., 2018). However, the authors mention that the exercise protocol in that study was intentionally chosen for its unique ability to suppress food intake based on a previously published article (Mani et al., 2018). This protocol also used high intensity exercise and electrical shock to stimulate running, which may have induced effects that wouldn’t be seen in a more physiologically relevant setting. In contrast, another independent study reported that only rats who exercised above their lactate threshold (>75% VO2_max_) had a significant increase in activation in the PVN and the ARC, compared to rats performing low-intensity exercise (Soya et al., 2011), further suggesting that neuronal activation is affected differently based on exercise intensity. However, in the aforementioned study, the specific subpopulations that were activated in ARC were not specified. In the present study, we used a similar exercise protocol of an estimated 75% VO2_max_, but for the first time utilized NPY-GFP reporter mice to specifically investigate ARC^AgRP/NPY^ neuronal activation. Consistent with previous findings (Soya et al., 2011; Lima et al., 2014), we found an acute bout of exercise resulted in both PVN and ARC neuronal activation. Taken together, the intensity of exercise seems to be key factor in determining the neuronal activation and associated feeding behaviors.

The paraventricular nucleus of the hypothalamus (PVN) is an important downstream site for the mediation of the ARC^AgRP/NPY^ regulation of energy homeostasis, with dense ARC^AgRP/NPY^ projections in this area (Wang et al., 2015). It has been reported that exercise promotes PVN neuronal activation (Lima et al., 2014). These evidences led us to suspect that exercise induced food intake may occur via ARC^AgRP/NPY^-PVN^SIM1^ circuitry. In agreement with a previous study (Soya et al., 2011), increased activation in PVN neurons immediately after exercise was observed. However, in this study, inhibition of the ARC^AgRP/NPY^ neurons during exercise had no effect on the activation of SIM1 expressing neurons in the PVN, despite the observed decreases in post-exercise food intake. This indicates that PVN^SIM1^ neurons are not downstream mediators of ARC^AgRP/NPY^ neurons during acute exercise-induced food intake. However, we cannot rule out the possibility of the opposite neurocircuitry in which exercise activates PVN^SIM1^ neurons to increase ARC^AgRP/NPY^ neuron activity. Recent studies have shown that the activation of subsets of PVN^SIM1^ neurons can markedly increase activation ARC^AgRP/NPY^ neurons and increase feeding in sated mice, thus indicating a reciprocal circuit may exist in feeding behavior (Krashes et al., 2014). Therefore, it is possible that PVN^SIM1^ neuronal activation mediates ARC^AgRP/NPY^ neuronal activation by acute exercise. In future studies, inhibition of these PVN^SIM1^ neurons through a SIM1-Cre transgenic mouse model could elucidate the causes of the changes in neuronal activation induced by acute exercise. Alternative neurons in the PVN that release excitatory synaptic input onto ARC^AgRP/NPY^ neurons to promote food intake are thyrotropin-releasing hormone (TRH)-expressing neurons or pituitary adenylate cyclase-activating polypeptide (PACAP) neurons (Krashes et al., 2014). Considering the complexity of the brain’s neurocircuitry, there are likely multiple neuronal mechanisms involved in the remodeling of synaptic of ARC^AgRP/NPY^ neurons in response to exercise.

In summary, this study demonstrates for the first time that a single bout of moderate intensity treadmill exercise acutely increases ARC^AgRP/NPY^ neuronal activation, while ARC^POMC^ neuron activation remains unaffected. Notably, this exercise induced energy deficit also causes an acute increase in food intake immediately post-exercise in an ARC^AgRP/NPY^ neuron-dependent manner. Thus, this data demonstrates exercise induced ARC^AgRP/NPY^ activation is critical in promoting food intake post-exercise. This association in ARC^AgRP/NPY^ neuron activation and food intake provide insight into the mechanisms promoting refueling and energy homeostasis after an exercise-induced caloric deficit.

## Data Availability Statement

The raw data supporting the conclusions of this article will be made available by the authors, without undue reservation, to any qualified researcher.

## Ethics Statement

The animal study was reviewed and approved by the Institutional Animal Care and Use Committee for the University of East Carolina, Greenville (Greenville, NC, United States).

## Author Contributions

WB, BL, and TL performed all the experiments. WB and HH analyzed the data and designed the experiments. PL, ZR, and TL helped on discussion. YY helped to maintain the experimental mice. WB, TL, and HH wrote the manuscript. All the co-authors reviewed and approved submission of the manuscript.

## Conflict of Interest

The authors declare that the research was conducted in the absence of any commercial or financial relationships that could be construed as a potential conflict of interest.

## References

[B1] Andermann M. Lowell B. (2017). Toward a wiring diagram understanding of appetite control. *Neuron* 95 757–778. 10.1016/j.neuron.2017.06.014 28817798 PMC5657399

[B2] Aponte Y. Atasoy D. Sternson S. M. (2011). AGRP neurons are sufficient to orchestrate feeding behavior rapidly and without training. *Nat. Neurosci.* 14 351–355. 10.1038/nn.2739 21209617 PMC3049940

[B3] Balthasar N. Dalgaard L. T. Lee C. E. Yu J. Funahashi H. Williams T. (2005). Divergence of melanocortin pathways in the control of food intake and energy expenditure. *Cell* 123 493–505. 10.1016/j.cell.2005.08.035 16269339

[B4] Baskin D. G. Breininger J. F. Schwartz M. W. (1999). Leptin receptor mRNA identifies a subpopulation of neuropeptide Y neurons activated by fasting in rat hypothalamus. *Diabetes Metab. Res. Rev.* 48 828–833. 10.2337/diabetes.48.4.828 10102700

[B5] Berger M. Hagg S. Ruderman N. B. (1975). Glucose metabolism in perfused skeletal muscle. Interaction of insulin and exercise on glucose uptake. *Biochem. J.* 146 231–238. 10.1042/bj1460231 807202 PMC1165292

[B6] Björntorp P. (1981). The effects of exercise on plasma insulin. *Int. J. Sports Med.* 2 125–129. 10.1055/s-2008-1034597 6277812

[B7] Broom D. R. Batterham R. L. King J. A. Stensel D. J. (2009). Influence of resistance and aerobic exercise on hunger, circulating levels of acylated ghrelin, and peptide YY in healthy males. *Am. J. Physiol. Regul. Integr. Compar. Physiol.* 296:R35. 10.1152/ajpregu.90706.2008 18987287

[B8] Chen C. Bechtold A. Tabor J. Bonham A. (2009). Exercise reduces GABA synaptic input onto nucleus *Tractus Solitarii* baroreceptor second-order neurons via NK1 receptor internalization in spontaneously hypertensive rats. *J. Neurosci.* 29 2754–2761. 10.1523/JNEUROSCI.4413-08.2009 19261870 PMC2682348

[B9] Dey S. Singh R. H. Dey P. K. (1992). Exercise training: significance of regional alterations in serotonin metabolism of rat brain in relation to antidepressant effect of exercise. *Physiol. Behav.* 52 1095–1099. 10.1016/0031-9384(92)90465-e 1283013

[B10] Gropp E. Shanabrough M. Borok E. Xu A. W. Janoschek R. Buch T. (2005). Agouti-related peptide–expressing neurons are mandatory for feeding. *Nat. Neurosci.* 8 1289–1291. 10.1038/nn1548 16158063

[B11] Group D. P. P. R. (2002). Reduction in the incidence of type 2 diabetes with lifestyle intervention or metformin. *N. Engl. J. Med.* 2002 393–403. 10.1056/nejmoa012512 11832527 PMC1370926

[B12] He Z. Gao Y. Alhadeff A. L. Castorena C. M. Huang Y. Lieu L. (2018). Cellular and synaptic reorganization of arcuate NPY/AgRP and POMC neurons after exercise. *Mol. Metab.* 18 107–119. 10.1016/J.MOLMET.2018.08.011 30292523 PMC6308029

[B13] Huang H. Kong D. Byun K. H. Ye C. Koda S. Lee D. H. (2012). Rho-kinase regulates energy balance by targeting hypothalamic leptin receptor signaling. *Nat. Neurosci.* 15 1391–1398. 10.1038/nn.3207 22941110 PMC3458121

[B14] Huang H. Lee S. H. Ye C. Lima I. S. Oh B.-C. Lowell B. B. (2013). ROCK1 in AgRP neurons regulates energy expenditure and locomotor activity in male mice. *Endocrinology* 154 3660–3670. 10.1210/en.2013-1343 23885017 PMC3776869

[B15] King N. A. Burley V. J. Blundell J. E. (1994). Exercise-induced suppression of appetite: effects on food intake and implications for energy balance. *Eur. J. Clin. Nutr.* 48 715–724. 7835326

[B16] King N. A. Hopkins M. Caudwell P. Stubbs R. J. Blundell J. E. (2008). Individual variability following 12 weeks of supervised exercise: identification and characterization of compensation for exercise-induced weight loss. *Intern. J. Obes.* 32 177–184. 10.1038/sj.ijo.0803712 17848941

[B17] Kissileff H. R. Pi-Sunyer Fx Segal K. Meltzer S. Foelsch P. A. (1990). Acute effects of exercise on food intake in obese and nonobese women. *Am. J. Clin. Nutr.* 52 240–245. 10.1093/ajcn/52.2.240 2375289

[B18] Knner A. C. Klckener T. Brning J. C. (2009). Control of energy homeostasis by insulin and leptin: targeting the arcuate nucleus and beyond. *Physiol. Behav.* 97 632–638. 10.1016/j.physbeh.2009.03.027 19351541

[B19] Krashes M. J. Koda S. Ye C. Rogan S. C. Adams A. C. Cusher D. S. (2011). Rapid, reversible activation of AgRP neurons drives feeding behavior in mice. *J. Clin. Invest.* 121 1424. 10.1172/jci46229 21364278 PMC3069789

[B20] Krashes M. J. Shah B. P. Madara J. C. Olson D. P. Strochlic D. E. Garfield A. S. (2014). An excitatory paraventricular nucleus to AgRP neuron circuit that drives hunger. *Nature* 507 238–242. 10.1038/nature12956 24487620 PMC3955843

[B21] Leibowitz S. F. Hammer N. J. Chang K. (1981). Hypothalamic paraventricular nucleus lesions produce overeating and obesity in the rat. *Physiol. Behav.* 27 1031–1040. 10.1016/0031-9384(81)90366-8 7335803

[B22] Lima P. M. A. Santiago H. P. Szawka R. E. Coimbra C. C. (2014). Central blockade of nitric oxide transmission impairs exercise-induced neuronal activation in the PVN and reduces physical performance. *Brain Res. Bull.* 108 80–87. 10.1016/J.BRAINRESBULL.2014.09.002 25234442

[B23] Lin H. V. Plum L. Ono H. Gutiérrez-Juárez R. Shanabrough M. Borok E. (2010). Divergent regulation of energy expenditure and hepatic glucose production by insulin receptor in agouti-related protein and POMC neurons. *Diabetes* 59 337–346. 10.2337/db09-1303 19933998 PMC2809966

[B24] Mani B. K. Castorena C. M. Osborne-Lawrence S. Vijayaraghavan P. Metzger N. P. Elmquist J. K. (2018). Ghrelin mediates exercise endurance and the feeding response post-exercise. *Mol. Metab.* 9 114–130. 10.1016/j.molmet.2018.01.006 29396372 PMC5870098

[B25] Mayer J. Marshall N. B. Vitale J. J. Christensen J. H. Mashayekhi M. B. Stare F. J. (1954). Exercise, food intake and body weight in normal rats and genetically obese adult mice. *Am. J. Physiol. Legacy Content* 177 544–548. 10.1152/ajplegacy.1954.177.3.544 13158612

[B26] Routh V. H. (2010). Glucose sensing neurons in the ventromedial hypothalamus. *Sensors* 10 9002–9025. 10.3390/s101009002 22022208 PMC3196991

[B27] Santoso P. Nakata M. Shiizaki K. Boyang Z. Parmila K. Otgon-Uul Z. (2017). Fibroblast growth factor 21, assisted by elevated glucose, activates paraventricular nucleus NUCB2/Nesfatin-1 neurons to produce satiety under fed states. *Sci. Rep.* 7:45819. 10.1038/srep45819 PMC5379189 28374855

[B28] Schefer V. Talan M. I. (1996). Oxygen consumption in adult and aged C57BL/6J mice during acute treadmill exercise of different intensity. *Exp. Gerontol.* 31 387–392. 10.1016/0531-5565(95)02032-2 9415121

[B29] Soya H. Okamoto M. Matsui T. Lee M. Inoue K. Nishikawa S. (2011). Brain activation via exercise: exercise conditions leading to neuronal activation & hippocampal neurogenesis. *J. Exer. Nutr. Biochem.* 6 1–10. 10.5717/jenb.2011.15.1.1

[B30] Stiegler P. Cunliffe A. (2006). The role of diet and exercise for the maintenance of fat-free mass and resting metabolic rate during weight loss. *Sports Med.* 36 239–262. 10.2165/00007256-200636030-00005 16526835

[B31] Tong Q. Ye C.-P. Jones J. E. Elmquist J. K. Lowell B. B. (2008). Synaptic release of GABA by AgRP neurons is required for normal regulation of energy balance. *Nat. Neurosci.* 11 998–1000. 10.1038/nn.2167 19160495 PMC2662585

[B32] Verger P. Lanteaume M. T. Louis-Sylvestre J. (1994). Free food choice after acute exercise in men. *Appetite* 22 159–164. 10.1006/appe.1994.1015 8037440

[B33] Wang D. He X. Zhao Z. Feng Q. Lin R. Sun Y. (2015). Whole-brain mapping of the direct inputs and axonal projections of POMC and AgRP neurons. *Front. Neuroanat.* 9:40 10.3389/fnana.2015.00040 PMC4375998 25870542

[B34] Wu Q. Boyle M. P. Palmiter R. D. (2009). Loss of GABAergic signaling by AgRP neurons to the parabrachial nucleus leads to starvation. *Cell* 137 1225–1234. 10.1016/j.cell.2009.04.022 19563755 PMC2729323

[B35] Xu Y. Jones J. E. Kohno D. Williams K. W. Lee C. E. Choi M. J. (2008). 5-HT 2C Rs expressed by pro-opiomelanocortin neurons regulate energy homeostasis. *Neuron* 60 582–589. 10.1016/j.neuron.2008.09.033 19038216 PMC2631191

[B36] Yaswen L. Diehl N. Brennan M. B. Hochgeschwender U. (1999). Obesity in the mouse model of pro-opiomelanocortin deficiency responds to peripheral melanocortin. *Nat. Med.* 5 1066–1070. 10.1038/12506 10470087

